# Around the Tables – Contextual Factors in Healthcare Coverage Decisions Across Western Europe

**DOI:** 10.15171/ijhpm.2019.145

**Published:** 2020-01-19

**Authors:** Tineke Kleinhout-Vliek, Antoinette de Bont, Meindert Boysen, Matthias Perleth, Romke van der Veen, Jacqueline Zwaap, Bert Boer

**Affiliations:** ^1^Erasmus School of Health Policy & Management, Erasmus University Rotterdam, Rotterdam, The Netherlands.; ^2^National Institute for Health and Care Excellence (NICE), London, UK.; ^3^Federal Joint Committee (Gemeinsamer Bundesausschuss), Berlin, Germany.; ^4^Erasmus School of Social and Behavioural Sciences, Erasmus University Rotterdam, Rotterdam, The Netherlands.; ^5^National Health Care Institute (Zorginstituut Nederland), Diemen, The Netherlands.

**Keywords:** Healthcare Decision-Making, Priority Setting, Contextual Factors, International Comparison, Western Europe

## Abstract

**Background:** Across Western Europe, procedures and formalised criteria for taking decisions on the coverage (inclusion in the benefits basket or equivalent) of healthcare technologies vary substantially. In the decision documents, which display the justification of, the rationale for, these decisions, national healthcare institutes may employ ‘contextual factors,’ defined here as* situation-specific* considerations. Little is known about how the use of such contextual factors compares across countries. We describe and compare contextual factors as used in coverage decisions generally and 4 decision documents specifically in Belgium, England, Germany, and the Netherlands.

**Methods:** Four group interviews with 3 experts from the national healthcare institute of each country, document and web site analysis, and a workshop with 1 to 2 of these experts per country were followed by the examination of the documents of 4 specific decisions taken in each of the 4 countries, sampled to vary widely in type of technology and decision outcome.

**Results:** From the available decision documents, we conclude that in every country studied, contextual factors are established ‘around the table,’ ie, in deliberation. All documents examined feature contextual factors, with similar contextual factor patterns leading to similar decisions in different countries. The Dutch decisions employ the widest variety of factors, with the exception of the societal functioning of the patient, which is relatively common in Belgium, England, and Germany. Half of the final decisions were taken in another setting, with the consequence that no documentation was retrievable for 2 decisions.

**Conclusion:** First, we conclude that in these countries, contextual factors are *actively integrated* in the decision document, and that this is achieved in deliberation. Conceptualising contextual factors as both situation-specific and actively-integrated affords insight into practices of contextualisation and provides an encouragement for exchange between decision-makers on more qualitative aspects of decisions. Second, the decisions that lacked a publicly accessible justification of the final decision document raised questions on the decisions’ legitimacy. Further research could address patterning of contextual factors, elucidate why some factors may remain implicit, and how decisions without a publicly available decision document may enable or restrain decision-making practice.

## Background

Challenging coverage decision dossiers on a specific healthcare technology may show up on desks placed in different countries around the same time.^1^ Within their varying healthcare systems, decision-makers are struggling with the exact same question: should our society pay for this?^2^ The processes to arrive at an answer to this question have not been aligned across Western Europe, though there are strong similarities. Western European systems generally utilise some form of health technology assessment for healthcare technologies,^3-6^ which include technologies as wide-ranging as (orphan) drugs and medical devices, and “have clear objectives reflected in reimbursement criteria.”^5^ A formalised set of reimbursement criteria may, however, not necessarily wholly capture all reasons for or against coverage provided in a (publicly available) coverage decision document,^7^ which may result in differences between countries that might not be expected based on their respective formalised criteria sets.^8-10^ When surveyed, decision-makers indeed frequently acknowledge the presence of additional elements that impact coverage decisions. Scholars group such additional elements under umbrellas like ‘contextual factors.’^3,10-16^

What this umbrella of contextual factors holds exactly has recently attracted scholarly interest for ‘decisions of value’ in the healthcare field.^17-20^ Williams et al initiated the discussion with a literature review focused on meso-level decision-making, exploring both ‘inner’ and ‘outer’ contexts and how these may affect such decisions of value.^14^ Many of the papers reviewed by Williams et al give ‘context’ as a relatively abstract explanation, and as something that is not necessarily visible in the final decision document. Examples from this review include organisational/institutional forces,^21,22^ political constraints,^23^ and economic climate or market forces.^24,25^ The field of Science and Technology Studies (STS), and Asdal specifically, challenges us to not use contexts as what she terms non-specific ‘explanatory resources.’^26,27^ Not content with doing away with context altogether either, Asdal encourages us to:

*“[Grasp] the events of your study as, literally, unique events. (…) [Consequently,] the situation as the context that needs to be ‘recovered’ is that which conditions or enables a specific utterance to happen.”* ^27^

In essence, she sees context as present in a specific decision situation, traceable to a specific moment in time: as *situation-specific*. This characteristic of situation-specificity, we argue, gives a handle on the contents of what Williams et al term “greater levels of judgement and intuitions” on the part of decision-makers.^14^ This paper follows suit in conceptualising contexts expressly not as external explanations for a situation and not as enduring backdrops of whatever kind, but rather as situation-specific entities that may be actively brought in by decision-makers. Moreover, we will term contexts that are present in the final decision documentation, which provides a justification, or rationale, for the decision, ‘contextual factors.’ In this, we narrow down and specify Williams et al’s original description of contextual factors.^17^ Of the contexts retrieved from literature by Williams et al, only 2 may be considered situation-specific, namely first, the information accessed by the decision-makers, or its absence, and especially high levels of uncertainty regarding this information; and second, the presence of specific interests impacting the decision. That the technology and its information would vary per decision may be considered logical; the latter finding resonates with situation-specific contexts identified elsewhere for macro-level coverage decisions.^13,28-31^

Although some of the papers reviewed by Williams et al describe minutely how the decision process unfolded, especially those included in the review by Vuorenkoski et al,^10^ little is known about how these contexts are integrated in the justification of, the rationale for, the decision specifically, as this often remains implicit in line with the surveys of policy-makers described above. To be precise, no studies have, to our knowledge, compared such use of contextual factors across countries (comparative papers primarily focus on the use of health technology assessment or related criteria such as cost-effectiveness and conclude that if decisions “vary,” they do so due to “additional factors”^8,32,33^). This paper compares how situation-specific contextual factors are integrated in coverage decision documents in 4 Western European countries. Contextual factors will be operationalised here by means of a list of previously-described necessity argumentations.^7^ Necessity argumentations have been shown to be used in coverage decisions around the world, but are not considered valid for every healthcare technology by everyone: their perceived validity varies per situation. These argumentations comprise a varied list, and include eg, the presence or absence of alternative treatments, the Rule of Rescue, the societal impact of (lack of) coverage, whether similar treatments are covered, medical necessity, and moral hazard considerations. Specifically, this research adds to existing literature by comparison of such contextual factor use in 4 widely varying healthcare technologies across 4 relatively similar countries in Western Europe, namely Belgium, England, Germany, and the Netherlands.

### Research Aim

This paper will explore situation-specific contextual factors in healthcare coverage decisions. We will answer the following main research question: how do Belgium, England, Germany, and the Netherlands use contextual factors in healthcare coverage decisions generally, and how does contextual factors use compare across 4 specific decisions? This study is divided into 2 parts. Part 1 examines how contextual factors are used in decision documentations generally through interviews and a workshop, enriched by document and web site analysis. Part 2 compares the contextual factors present in 4 specific decisions: nivolumab (Opdivo^®^), benzodiazepines, smoking cessation therapy, and walking aids with wheels, taken in each of the 4 countries by examining the relevant decision documents.

## Methods

### Approach

This study describes the outcomes of an international research collaboration of decision-makers or policy advisors employed at 4 national healthcare institutes: National Institute for Health and Care Excellence (NICE) in England; the German Federal Joint Committee (*Gemeinsamer Bundesausschuss* , G-BA); the Healthcare Knowledge Centre/National Institute for Health and Disability Insurance (HCKC/NIHDI) in Belgium; and the Dutch National Health Care Institute (*Zorginstituut Nederland* , ZIN) in the Netherlands with researchers from the Erasmus School of Health Policy & Management and the Erasmus School of Social and Behavioural Science, Rotterdam, the Netherlands on healthcare coverage decisions. For further information on the national healthcare institutes, please see section ‘working procedures’ in Supplementary file 1. We provide an analysis of contextual factors in coverage decision documents generally (part 1 of the study) and in 4 specific cases (part 2) in these 4 countries.

We have chosen to compare countries in the understanding that comparative research need not be a linear-causal exercise, meaning here that a specific attribute of the country need not be offered as the explanation for an observed phenomenon.^34,35^ Rather, this method was chosen because careful comparison of arguments and statements is thought to result in clearer definitions of these and more succinct questions for future research to address.^35^ Specifically, we started with semi-structured, active interviews^36^ in groups. We enriched the interview data by document and web site analysis as well as an extensive member check (during the workshop, see below).

We have opted to further extend the data gathered in part 1 with case studies from the 4 countries.^37,38^ The reason for doing so is that comparison between cases that are similar (decisions on the same healthcare technology) taken in varying situations (the different countries) aids generating insight especially where valuation processes (cf. decisions of value^14^) are concerned.^39^ In addition, the cases represent maximum variability.^40^ In this way, by comparing the countries’ general decision-making processes with 4 decisions specifically, we have chosen a small ‘string of comparisons,’ which has the potential to bring stronger clarification.^34^

To operationalise these contextual factors, we will employ necessity argumentations as our sensitising concepts.^41^ Necessity is an umbrella term that encapsulates disease severity and need-related argumentations, which are situation-specific because they vary strongly per technology examined, and explicated and integrated by virtue of being used in decision outcomes. Necessity argumentations comprise patient-specific considerations, such as above-mentioned disease severity, medical necessity and need, but also dignity, human rights and impact on societal functioning, and whether the condition the patient suffers from may be construed as ‘normal experience.’ Related are the argumentations related to the patient population, such as whether the technology will be relevant for a small number of patients. Other considerations have to do with the technology and its availability, such as the presence or absence of alternative treatment and coverage of similar treatments, but also moral hazard (over-usage) considerations. Another type of argumentation is society-related and concerns argumentations like societal (or individual) responsibility and impact of (lack of) coverage on wider society.

Necessity argumentations are used to provide a justification of, a rationale for, coverage decisions in several European countries, including France, Germany, Sweden, the United Kingdom, and the Netherlands.^7^ These argumentations are largely considered valid not only by decision-makers, but also by the public. In the overall study, the Netherlands was taken as the entry point in terms of country selection and overall study focus, because necessity has been described and used as a criterion for Dutch coverage decisions.^42,43^ England, Germany and Belgium were selected as comparator countries because of their similarities in terms of ‘healthcare system objectives’ such as equity and affordability, but also transparency in decision-making.^5^ This was expected to result in a publicly available decision, enabling our data analysis.^34^ The countries’ specific working procedures for decision-making may be found in Supplementary file 1.

### Data Collection

The data collection consisted of 2 parts corresponding to the 2 parts of the study: first, a round of semi-structured group interviews with experts, general policy document and web site analysis and a workshop with all the authors (for an overview, see Table 1); and second, document analysis of the 4 decisions taken across the 4 countries (for an overview, see Table 2).

**Table 1:** Overview of Interviews and Workshops Comprising Part 1 of the Study

**Interview Date**	**Description**
February 24, 2017	Appraisal committee ZIN group interview (RvdV, JZ, and a colleague)
March 17, 2017	HCKC/NIHDI group interview (RM and 2 colleagues)
April 6, 2017	G-BA group interview (MP and 2 colleagues)
June 23, 2017	NICE/NHS-England group interview (MB and 2 colleagues)
October 18, 2017	Additional interview (RvdV)
December 13, 2017	Workshop at ZIN

Abbreviations: ZIN, Zorginstituut Nederland; HCKC/NIHDI, Healthcare Knowledge Centre/National Institute for Health and Disability Insurance; G-BA, Gemeinsamer Bundesausschuss; NICE, National Institute for Health and Care Excellence; NHS, National Health Service.

**Table 2:** Overview of Documents Analysed Comprising Part 2 of the Study

**Decision**	**Country**	**Document**
Nivolumab	The Netherlands	Package advice nivolumab (Opdivo®) including letter to the Minister of Health, Welfare, and Sports, dated December 8, 2015^44^
	Belgium	Evaluation report day 90, Dossier 736 and 737, Second request report, dated July 6, 2016^45^
	England	NICE Technology appraisal guidance: Nivolumab for previously treated squamous non-small-cell lung cancer (TA483), dated November 1, 2017^46^
	Germany	File for Benefit Assessment in accordance with section 35a of SGB-V, nivolumab (Opdivo^®^), dated April 28, 2016^47^
Benzodiazepines	The Netherlands	Package advice 2009, Publication number 274, dated April 3, 2009^48^ Letter to the Minister of Health, Welfare, and Sports, Reimbursement benzodiazepines, dated April 21, 2008^49^
	Belgium	-
	England	Generalised anxiety disorder in adults, the NICE guideline on management in primary, secondary and community care^50^ Generalised anxiety disorder and panic disorder in adults: management. Clinical guideline [CG113], dated January 2011^51^
	Germany	-
Smoking cessation therapy	The Netherlands	Help with smoking cessation: insured care?, dated June 30, 2008^52^ Smoking cessation programme: insured care! Publication number 276, dated April 21, 2009^53^
	Belgium	Effectiveness and cost-effectiveness of treatment for smoking cessation, HCKC, dated 2004^54^
	England	Stop smoking interventions and services, NICE guideline (NG92), dated March 28, 2018^55^
	Germany	Regulation exclusion of medicines for heightening quality of life in accordance with Section 34 (1) Sentence 7 SGB V (Lifestyle Drugs), Annex II to Section F of the Medicine-Directive, dated January 28, 2017^56^
Walking aid with wheels	The Netherlands	Report Medical aids 2010, Publication number 286, dated April 2, 2010^57^
	Belgium	Memorandum main working group number 2003/6.4, Main group 1.4, Walking Aids Adults, dated July 14, 2003^58^
	England	https://www.nhs.uk/conditions/social-care-and-support-guide/care-services-equipment-and-care-homes/walking-aids-wheelchairs-and-mobility-scooters/, dated August 8, 2018
	Germany	GKV-Spitzenverband Update of the product group 10 "Walking aids" of the aid directory according to Section 139 SGB- VvomAf, dated August 27, 2018^59^

Abbreviations: HCKC, Healthcare Knowledge Centre; NICE, National Institute for Health and Care Excellence; SGB-V, German Social Code, Book 5; GKV, National Association of Statutory Health Insurance Funds. Note: For the English Benzodiazepines decision, please note that the second document analysed (Clinical guideline 113) is based on the former document.

Part 1 of the data collection aimed to answer the first research question, was designed by authors AdB, BB, and TKV, and consisted of 4 semi-structured interviews, which were conducted in 2.5-3 hour conversations at each of the 4 healthcare institutes. These semi-structured interviews were conducted by BB, JZ, and TKV and held with 1 national expert, who acted as the primary point of contact per institute and subsequently agreed to co-author this paper (MB, MP, and RvdV/JZ) and 2 colleagues, ie, 3 interviewees per country, with the exception of Belgium, who opted out of authorship. These national experts hold the following roles: former Head of HCKC, Director of the Centre for Health Technology Evaluation at NICE, Head of the Department of Medical Advice at G-BA, and the Chair and Secretary of the Appraisal Committee at ZIN.

An exception was the Dutch group interview, where 2 experts agreed to co-author this paper and only 1 additional colleague was present, and RvdV was interviewed 1 additional time by TKV (see Table 3). During the interviews, the interviewees all offered a deep understanding of their country’s healthcare decision processes and the use of contextual factors therein through giving a presentation on their decision-making procedure. This presentation was prepared beforehand, the interviewee giving the presentation was asked to provide information on the general coverage decision-making process in their country and its institutional bedding. They were also aware that the interviewers were interested in necessity argumentations in particular, and thus provided information on these types of considerations as well. This was followed by a presentation by TKV on what the interviewers understood as contextual factors (that is, necessity argumentations) and an extended group interview on the role of these types of factors on the decision-making process generally. The questions used to guide these interviews may be found in Supplementary file 2. The 4 semi-structured group interviews were audio-recorded, transcribed, and coded for necessity argumentation use. These analyses were enriched by analysis of relevant policy documents and websites as offered by the interviewees. This was generally achieved by perusing the general websites of the respective institutes and documents pertaining to their working procedures in case of lack of clarity.

**Table 3:** Overview of Contextual Factors Operationalised as Necessity Argumentations and Their Respective Descriptions^7^ Combined With Information on Which Factor Was Used in Which Decision in Which Country

**Contextual Factor**	**Description**	**Nivolumab**	**Benzodiazepines**	**Smoking Cessation Therapies**	**Walking Aids With Wheels**
Definition of Illness	Whether the ailment is considered an illness for which treatment is necessary	NL		NL, BE, EN, DE	
Equity/Fairness/Justice	Whether coverage would be necessary to counter injustice/inequity/lack of fairness in (access to) treatment			NL, EN	
Individual Cost	Whether lack of coverage would stop patients from buying necessary care themselves due to prohibitive cost	NL	NL	NL	NL
Individual Responsibility	Whether the individual is considered responsible for paying for this treatment			NL	NL
Medical Necessity	Whether or not a treatment is considered to be “medically necessary” or a “medical necessity”		NL		
Morbidity/Severity	Whether the physical and/or psychosocial morbidity associated with a certain ailment constitutes such a need that coverage is considered necessary	NL, BE, EN, DE	NL, EN	NL, BE, EN	BE, EN, DE
Need	The extent to which the patient is considered to be in need for which treatment is necessary	NL, BE, EN, DE			BE
(No) Alternative	Whether or not viable alternatives are considered to be present which would make coverage more or less necessary	NL, BE, EN	NL, EN		DE
Patient-Diagnosis	Whether an illness is self-reported rather than diagnosed by a doctor		NL, EN		
Range of Normality	Whether the experience of the patient is considered normal or abnormal to such an extent that coverage is deemed necessary				NL
Similar Treatments	Whether similar treatments are covered or not (meaning that this *type* of treatment is considered necessary)			NL, DE	
Societal Impact	Whether coverage is considered necessary to allay the impact this disease has on people beyond the patient			NL, BE	
Societal Functioning	Whether coverage would aid a person’s necessary functioning in society	BE, EN	EN	NL, DE	BE, EN, DE
Vulnerability/Compassion	Whether a compassionate response to vulnerable groups, eg, children, in the form of coverage is considered to be a necessity			NL, BE	
Substitution	Whether other (eg, heavier dosage or more expensive than necessary) medicines or care would be consumed or used by patients as a result of a negative coverage decision		NL		
Under-consumption	Whether less medicines or treatments than necessary would be consumed or used by patients as a result of a negative coverage decision (the direct opposite of ‘Moral Hazard’)			NL	

Abbreviations: NL, the Netherlands; BE, Belgium; EN, England; DE, Germany. *Note:* The argumentation types that were not present in this data set (namely, Dignity, Human Right, Moral Hazard, Rule of Rescue, Small Number of Patients, and Societal Responsibility) were omitted from this table.

The findings gathered during the group interviews (supplemented by the information from the web site and document analysis) were presented by TKV at a one-day workshop, held in the Netherlands, attended by all authors (December 13, 2017). The explicit goal of this workshop was to gain deeper insight into the use of contextual factors in the different countries including similarities and differences as well as to provide a member check on the collected data. In addition, several analytical angles were discussed, and the decision to add part 2 of the research was made. Part 2’s aim was to examine the final documentation pertaining to 4 decisions taken across the 4 countries of interest. Three inclusion criteria for the case studies were formulated:

1. Necessity argumentations feature prominently in the final documentation of the coverage decision in the Netherlands;
2. The coverage decision outcome varies in the 4 countries, with at least 1 outcome deviating from the rest;
3. The cases together represent maximum variation in the patterns of use of the argumentation types.^7^

For part 2, then, policy-makers employed at ZIN (JZ and a colleague) aided with choosing 8 cases that fulfilled the first criterion and that were sufficiently different in type of technology. Inclusion criteria numbers 2 and 3 narrowed the list down to 4 cases: nivolumab (Opdivo^®^), benzodiazepines, smoking cessation therapy, and walking aids with wheels. Of the 16 decisions (4 countries times 4 health technologies), only 14 yielded a document to analyse, and 3 decisions yielded 2 documents, to a total of 17 documents. Approximately half were obtained through website searches of the relevant institutes, with the other half contributed by experts or colleagues working at that country’s healthcare institute (for an overview of the documents analysed, see Table 2). TKV was aided by a student in analysing all coverage decision documents for the primary arguments separately, again using the list of necessity argumentations (Table 3) as codes, using good language skills in Dutch (for the Dutch and Belgian documents) and English and sufficient aptitude in reading German, aided by online translation and where needed by professionals who were directly involved in the coverage decision processes. The coding was done by TKV by specifically searching for forms of these 20 necessity argumentations. The outcomes were discussed by AdB, BB, and TKV in several iterative meetings, and all other collaborators commented on the resulting analysis and agreed upon the final text.

## Results

This section provides an overview of how contextual factors, operationalised as necessity argumentations, generally play a role in each country, followed by a section on the use of contextual factors in the 4 case studies. A short introduction to the decision process in the different countries (derived from the website and policy document analysis and the presentation given by the interviewees during the semi-structured interviews) may be found in Supplementary file 1.

### Part 1: Use of Contextual Factors in General

From the presentations during the semi-structured group interviews in each of the 4 countries, as well as from the policy document and web site analysis, we conclude the following after introducing the interviewees to the list of necessity argumentations, sensitising them to the topics.

According to the interviewees, their respective national healthcare institutes all use necessity argumentations as contextual factors in their decisions in addition to the formalised criteria (outlined below). Members of the research team easily provided examples where, in their eyes, a wide variety of these non-formalised, situation-specific contextual factors were employed. These examples included, but were not limited to: inclusion of smoking cessation (Belgium, see case study below); exclusion of homeopathy (England, decided by the National Health Service [NHS], perceived by interviewee as a necessity argumentation); exclusion of over-the-counter medicines (England, due to low cost per patient); positive “Nikolaus decision” (Germany, due to a small number of patients); exclusion of immunisation for travel in free time (Germany, this falling under personal responsibility). Notably, the workshop attendees underlined that not all these argumentations might be identifiable in the final decision: sometimes these decisions were, apparently, ‘gut decisions,’ with reasons remaining implicit.

More generally, reflecting on the list of contextual factors, the Belgian interviewee stated:

*“[Contextualisation] will remain discursive all the time”* (workshop, 13.12.2017).

This observation highlighted that contextualisation is left open to be established in argumentations in a deliberative setting: ‘around the table.’ It also accentuated the difference between contextual factors, which are situation-specific, and formalised criteria, which are not. This does not mean that of all the necessity argumentations, none have been formalised into criteria. In fact, the national healthcare institutes have all formalised the use of these contextual factors, but to different extents.

Least formalised is Belgium, where no list of stringent criteria exists: rather, decisions are made, and contextual factors formulated, by different working groups. Some necessity argumentations are explicitly reflected in the criteria used in the Unmet Needs Programme, which features therapeutic need (discomfort, life expectancy, quality of life) and societal need (budget impact, incidence/prevalence), but this list is not widely used for decision-making.^60^

In England, the situation could be called most intricate. NICE general procedure turns on clinical and cost-effectiveness, with thresholds for opportunity costs. Disease severity is formally included in the application of the ‘end-of-life’ criteria, which allows additional flexibility only where a patient’s life expectancy is less than 2 years. It is also used in the deliberative decision-making process where the independent committee has the flexibility to accept cost-effectiveness estimates that are higher than what would otherwise be considered value for money. Moreover, the NICE pre-selection procedure for highly specialised technologies employs elements like chronicity and acute death as criteria.

For Germany, ‘necessity’ is 1 of 3 formalised criteria described in the German Social Code, Book 5 (SGB-V). However, it has not been specified to a great extent; the criteria in Germany are considered “generally formulated.”^61^ Disease severity does influence the level of evidence required in G-BA’s decisions (varying from anecdotal evidence to randomised controlled trials).

The country with the highest formalisation is the Netherlands; ZIN operationalises necessity as 1 of 4 formal package criteria (next to effectiveness, cost-effectiveness, and feasibility). This is explicated in their formal documentations as disease severity, or individual burden of disease, and individual cost considerations.^62,63^

### Part 2: Use of Contextual Factors in 4 Case Studies

The 4 decisions to be examined across the 4 countries, namely nivolumab, benzodiazepines, smoking cessation therapies, and walking aids with wheels, were selected per the criteria mentioned above, the documents analysed per decision are available in Table 2. Table 3 gives an overview of the used contextual factors per decision document per country.

#### Case Study 1: Nivolumab

All 4 countries decided on reimbursement of nivolumab for treatment of previously-treated squamous non-small-cell lung cancer.

The expectation of a large budget impact necessitated placing nivolumab in the Dutch package lock, awaiting an advised decision by ZIN and, potentially, subsequent price negotiations with the manufacturer.^64^ The Institute did indeed advise the Minister of Health against including nivolumab in the basic benefits package unless a price reduction of at least 40% could be achieved: the treatment was considered effective, but not cost-effective enough. In their decision documentation, the Institute argued that: “the burden of disease is high” as it is considered “a non-curative disease” with “limited life expectancy.” Interestingly, an alternative treatment (docetaxel) is mentioned in the pharmaco-economic report. Moreover, “the costs for the treatment is so high patients cannot be expected to pay.”^44^ The Minister did negotiate, and although the final price has not been made public, nivolumab is now available on the Dutch benefits package.

For Belgium, the official documentation retrieved is the ‘day 90’ report from the Committee for ‘advance compensation’ for Pharmaceuticals, and specifically the ‘second request’ document, which gives the latest insight into motivation concerning reimbursement and the details of the final coverage arrangement for nivolumab. The necessity argumentations visible are “weakness and fatigue, coughing, shortness of breath, pain” and a median survival number. Moreover, considerations around the quality of life feature in the comparison with doxacetel.^45^ This resulted in a positive decision.

As for ZIN, NICE’s Technology Appraisal Committee considered nivolumab effective but not cost-effective enough. Moreover, the uncertainty of the evidence was deemed too great. For these reasons, additional characteristics were considered: in the social value judgement several necessity argumentations were brought to the fore. The committee noted a high need due to lack of alternatives, with a high morbidity, a low life expectancy, and “symptoms which are difficult to manage.”^46^ This resulted in a recommendation of funding, but *only* through the Cancer Drugs Fund, which happened after a renegotiation on the price of nivolumab with manufacturer Bristol-Myers Squibb.^65^

In Germany, drugs are covered with market entry, followed by a decision on additional benefit by G-BA, which may be used in turn for price negotiations. In the case of nivolumab, the Institute for Quality and Efficiency in Health Care (*Institut für Qualität und Wirtschaftlichkeit im Gesundheidswesen*) provided evidence concerning mortality, morbidity, quality of life, and several adverse event categories, noting considerable added benefit on all counts for non-small-cell lung cancer patients on nivolumab to benefit the decision made by G-BA. In terms of necessity argumentations, the report mentions the severity of the disease, the low absolute 10-year survival rate and the “need for drugs.” The Individual Cost is mentioned but not clearly as an argumentation.^47^ This was sufficient grounds not to exclude nivolumab from coverage.

#### Case Study 2: Benzodiazepines

In the Netherlands, ZIN advised the Minister of Health against continued coverage of benzodiazepines.^48,49^ The primary reasoning was that although the indication was for short-term use, prescriptions for benzodiazepines often ended up to be for chronic use. The Institute recommended improving the guidelines, but consultation with field parties revealed difficulties in defining eligibility criteria. Therefore, even despite the disease severity mentioned and the fact that short use of benzodiazepines following the applicable guidelines may be medically necessary (but chronic use not), the large amount of chronic users, the possibility that denying coverage would lead to patients choosing another, covered, medicine and the fact that the cost for 1 episode of benzodiazepines is approximately €12-16 were decisive in the negative coverage decision. The exception was made for 3 clearly defined indications: epilepsy, anxiety disorders, and multiple psychiatric disorders.

For the Belgian case, treatment of benzodiazepines is not covered by the health insurance. NIHDI has never appraised benzodiazepines as they have never received a request to do so.

In England, NICE guidelines recommend short-term use of benzodiazepines for several medical indications, which have separate appraisal documents. We analysed the case of adult generalised anxiety disorders which highlights several risks for general practitioners prescribing benzodiazepines, which fall under necessity argumentations. First, emphasis is laid on the high frequency of over-use due to tolerance and dependence. The documentation does highlight that use is only recommended in case of non-response to other medicines, which can be classed as an argumentation in favour of coverage. People with generalised anxiety disorder are described to have “long-standing and often uncontrollable worries and negative thoughts” which affect their “many areas of their lives, particularly relationships, self-esteem, daily activities, employment, work life and education.”^50,51^

G-BA has, like NIHDI, never received an appraisal request for benzodiazepines, but in contrast to Belgium, benzodiazepines are covered by the German sickness funds. The reason is that in Germany all drugs are covered after market entry except when they are specifically excluded either by law in the SGB-V or by G-BA. A systematic assessment of additional benefit (for the sake of price negotiations) was only established in 2011 by the Act on the Reform of the Market for Medicinal Products. Benzodiazepines were on the German market in 2010 already and are thus part of a “historical benefit package.”

#### Case Study 3: Smoking Cessation Therapies

This analysis is of decisions for both psychotherapeutic and pharmaceutical smoking cessation therapies.

In the Netherlands, ZIN dealt with several iterations of the coverage decision for smoking cessation therapies: they were covered, then no longer covered, and then covered again. One reason is that it became a political issue with changes in government affecting the reimbursement status. The final advice to the Minister has been to cover stop advice, intensive forms of interventions for behavioural change, and nortriptyline, but not several other interventions. The final decision hinged, at least partially, on the effectiveness: in essence, the health gain even a few extra successful stop attempts would yield, also visible in the argumentation that “a smoking cessation programme can reduce the damage caused by smoking to others.” In this extensive decision-making process, several necessity argumentations played a role.^53,54^ First, the individual is personally responsible for his health, and the costs for the individual were low, reducing the perceived necessity of coverage. Even so, there were indications that more people would attempt to quit smoking if it was covered, and especially those with a lower socio-economic status. Moreover, smoking was defined as an addiction and bad for health, leading to quality of life loss, causing damage to others, including unborn children and infants, treatment for which is usually covered by the basic benefits package.

Smoking cessation therapies have never formally been discussed by the committee for Reimbursement of Medicines at Belgian NIHDI. Not the mutualities, but the Belgian National Cancer Initiative covers €20 of the first 8 sessions of smoking cessation therapies^66^and from January 1, 2017, smoking cessation aid by a tobaccologist is reimbursed in Flanders.^67^ As in the Netherlands though, these therapies have not always been covered for everyone. The compulsory health insurance covered smoking cessation therapies for all pregnant women, revealing a potential Societal Impact argumentation, though this was rarely used. In 2004, HCKC published a report that all smoking cessation therapies are cost-effective that informed what is now a primary part of the National Cancer Initiative. This report was not a coverage decision, but analysis yielded that smoking was defined as a habit, strong descriptions of the morbidity caused by smoking, including the increased risk of having a baby with low birth weight.^54^

In England, smoking cessation therapies are available to all citizens over 12 years of age, for which NICE most recently published guidelines in 2018. These highlighted that “smoking is the main cause of preventable illness and premature death” and showed that all smoking cessation therapies were cost-effective enough to stay below the QALY threshold. The guidelines underscore that there are social inequalities in terms of tobacco use, which “make a significant contribution to inequalities in health.”^55^

Germany is the only of the 4 countries analysed in this project that does not cover smoking cessation therapies, as they are excluded from the benefits package by law (SGB-V §34). The title of the analysed documentation reveals they are considered “lifestyle medicines” aimed at improving quality of life, but no additional contextual factors are visible in the documentation.

#### Case Study 4: Walking Aids With Wheels (Rollators)

We analyse walking aids with 2, 3 or 4 wheels, also called rollators, for adults.

In the Netherlands, the Minister of Health was advised by ZIN to no longer include mobility aids in the basic benefits package, and 2 necessity argumentations were given for this. First, the cost for walking aids with wheels, crutches and many more walking aids was considered too low and secondly, “a walker with wheels is for common use,” meaning that it was, in Dutch society, considered normal to need one at a certain age.^57^

The Belgian NIHDI has published prescription guidelines which cover walking aids with wheels for those unable to stand up independently or safely.^58^

In England, this intervention has not been considered by NICE, but the NHS loans walking aids with wheels for free, after assessment by a physician. No specific appraisal documents were available for walkers with wheels, as they are only discussed in guidelines for specific conditions, which only specify that “you or someone you know” needs to have “difficulty walking or getting around (mobility).”^68^

Despite the fact that G-BA has not appraised walking aids with wheels specifically, they are available to all German citizens from their insurers upon indication: anyone experiencing reduced physical mobility or loss of balance is considered eligible, as long as other mobility aids have proved insufficient. This list is managed by the National Association of Statutory Health Insurance Funds (*GKV-Spitzenverband*).^59^

### Comparison of Contextual Factor Use in the 4 Cases

To start, across these cases, similar patterns in contextual factor use lead to similar decision outcomes. This is visible in 5 instances. First, there are strong similarities between the decisions for nivolumab in Belgium and Germany, which both strongly rely on the severity of the disease and patients’ quality of life and need, as well as the low survival rate. The other 2 decisions for nivolumab are highly reminiscent of these argumentations, but the Dutch and English both used them as input for price negotiations (which means the initial decision was negative). England and the Netherlands also strongly overlapped in their argumentation pattern for benzodiazepines, invoking the large amount of chronic users or over-usage as a reason to limit coverage, while acknowledging the difficulties experienced by the patient as well as the lack of alternatives. Fourth, there are some similarities between the Dutch and English decision for smoking cessation therapies, too, though the Dutch decision-making process featured a far greater number of contextual factors. Finally, the Belgian, English, and German decisions for walking aids with wheels also show strong overlap: if you are unable to stand up safely in these countries, a walking aid with wheels is provided for in some way.

Moreover, it would seem that the number of contextual factor types considered valid in one or more of the cases varies between the countries: the Belgian decisions feature 7 types, the English also 7 types, the German decisions 6 types, compared to 16 types of contextual factors present in the Netherlands. This shows that the Dutch decisions generally feature a high amount of contextual factors in this data set.

Finally, half the (final) decisions were not taken by the institutes analysed. This pertains to the Belgian and German benzodiazepines decisions, the Belgian and German smoking cessation therapies decisions, the English and German walking aids with wheels decisions and, to an extent, the Dutch nivolumab decision as it differed from the initial ZIN advice, and the English nivolumab decision as it was covered through the CDF rather than a positive decision by NICE. Many of these did, however, yield a document or web site to analyse (only 2 benzodiazepines decisions lacked such a decision document).

## Discussion

Contextualisation has recently been a raised as an important topic of interest for both policy practice and research regarding healthcare ‘decisions of value.’^14^ Our cross-country research team has defined contextual factors as *situation-specific* considerations, following Asdal.^27^ This enabled us to examine the coverage decision processes in 4 Western-European countries: Belgium, England, Germany, and the Netherlands, and specifically, to establish where contextual factors are used, both generally (part 1 of the study) and in 4 decisions taken across the 4 countries specifically (part 2). We have operationalised these situation-specific contextual factors using a list of previously-described necessity argumentations that are used across Europe and generally vary per decision.^7^

We draw the following conclusions. From part 1 of the study, we conclude that situation-specific contextual factors, operationalised as necessity argumentations, are present in decision-making processes at HCKC/NIHDI in Belgium; NICE in England; G-BA in Germany; and ZIN in the Netherlands. Some necessity argumentations have been formalised into criteria to be used for every decision in theory (individual burden of disease in England, Germany, and the Netherlands, and individual cost considerations in the Netherlands).^62,69-71^ This may raise the question whether these would fall outside the definition of contextual factors as situation-specific – however, our data show that not every decision analysed uses these formalised contextual factor(s) in practice. Specifically, expert interviewees underline that contextual factors are determined in deliberation, ‘around the table’: a setting present in some form at every institute studied.^61^ They offered many examples of specific decisions in which such factors were considered, though underlining that not all of these factors had been explicated in their respective documentations.

From part 2, we conclude that similar patterns in contextual factor usage lead to similar decisions in the countries studied (Belgium and Germany for nivolumab; England and the Netherlands for nivolumab and benzodiazepines; and Belgium, England and Germany for walking aids with wheels). This is an important conclusion, which may serve to encourage exchange between decision-makers in different countries on more qualitative aspects of coverage decisions in addition to the current collaborations on the more quantitative aspects. However, the decisions are still sufficiently different (in fact, they were explicitly selected as having different outcomes across the 4 countries) to preclude much more than exchange. The use of contextual factors in decisions, we would argue, would need to remain at the discretion of local decision-makers. In this data set, Dutch decisions employ the widest variety of contextual factors, and most often employ an argumentation type no other country employs. This with the notable exception of the societal functioning of the patient, which is a common consideration in Belgium, England, and Germany. This shows that in a sense, the argumentations are not 100% situation-specific. Instead, they are part of typifications, and these types are identifiable across situations, but not consistently. It is thus the *pattern* of contextual factors that is truly situation-specific.^29,72^ Future research could further address this idea of patterning, and how it influences coverage decisions. Finally, for all 4 institutes some decisions in this data set are taken or retaken by another actor, which often sometimes means that the documentation for the final decision is not publicly available.

Taking a step further in conceptualising contextual factors, we conclude that they are explicated around the table, in deliberation.This establishing around the table is the first element: we also conclude that contextual factors need to be *actively integrated* in the decision documentation, as not all factors established in deliberation seem to be present in the document.  Many scholars have in fact described, often based on interviews, that many such contextual factors remain implicit, either in both the deliberation and the documentation, or in the documentation only. This is perhaps to be expected, as localised processes of meaning-giving by the decision-makers themselves are described as implicit.^73,74^ We conclude, as Mann puts it, that contextualisation is an intervention,^75^ and as such, an active, situated process. As a consequence, this conceptualisation of contextual factors attunes us to effect that decision-makers have on how decisions are justified, and the expertise they bring to bear therein.The fact that these differences in explication of factors exist is of particular interest as it provides more insight into the how and why of evidence-informed deliberative processes.^76,77^ Further research should address why and how some factors remain implicit, whereas others are not only explicated but actively integrated in the final text.

Many of the documentations that should provide a justification/rationale for the final decision were, in fact, absent, and an alternative document was analysed if present. For some, we analysed the pre-final decision (nivolumab for England and the Netherlands, both negative decisions), in other cases, the decision was not taken by the national healthcare institute (walking aids with wheels for England and Germany), in others again, no decision was visible at all. This underscores that healthcare coverage decision-making is a process that involves many people in many places. It is a question of definition (‘does a decision on technology X fall under the remit of our national healthcare institute?’) but also a question of agenda setting (eg, NIHDI and G-BA had never received a request to appraise benzodiazepines). This backdrop to the decision is something that is not explicitly integrated into the documentation, but is definitely actively shaping the final decision.

This high prevalence of decisions for which the final documentation is not available is a particularly salient finding, as this highlights that the transparency of some decisions may be limited. Because these (final) decisions are made in another setting, the argumentations underlying the decision remain unknown. This is intriguing as many of the institutions studied are seeking to make processes more transparent in pursuit of increasing the legitimacy of their decisions.^5^ A more transparent decision is considered to heighten the legitimacy of this type of public decisions in general.^78-85^ Moreover, many authors hold that the coverage decision process should be based on consistently-applied, formalised criteria, visible in the documentation pertaining to the decision, and that having highly formalised criteria would potentially enable more rational, better-justifiable decisions.^86-90^ Yet, the Dutch nivolumab decision in particular demonstrates that having highly formalised criteria, as is the case in the Netherlands, does not preclude this particular type of decision-making.Further research may carefully consider the ways in which these ‘invisible’ decisions enable and restrain deliberative coverage decision-making practice, and how this relates to the legitimacy of these decisions.^91^

### Strengths and Limitations

Our paper covers a vital topic as it successfully visualises the contextual factors employed in coverage decisions generally and 4 decisions specifically in 4 countries. This paper does so without resorting to using general healthcare system characteristics as an explanation but instead seeks to draw more specific conclusions. Another strength of the paper lies in the methodology, and especially the case study selection, which was both grounded empirically through expert interviews and enriched by theoretical interest through the formulated criteria.

As the Netherlands was taken as the entry point for this study, both in terms of content as in terms of the place of work and residence of the majority of the authors, this will have had a significant impact. In particular, this is visible first, in terms of the case selection (as Dutch interviewees were asked for the initial list of potential cases); second, in the level of detail acquired in the case descriptions, which is relatively high for the Dutch decisions. It is also likely that it may have affected the research question itself. Asking questions concerning formalisation, or operationalisation, of criteria may be considered a typically *Dutch* pursuit, as visualised by the fact that the Netherlands has a rich history in terms of seeking to explicate decision criteria in this context.^42,69,71^

The use of necessity argumentations has narrowed the subject down content-wise to considerations that are likely to be present in decision documents. Future research could investigate how what remains implicit impacts decision-making. It is also important to note that the decisions studied span the last fifteen years, and it is likely that considerations are weighed or valued differently across that time span. Future research could address how the use of contextual factors may change over time.

As Bærøe noted,^17^ there is a difference between approaches aiming to formulate a comprehensive list, and those that would hold that this is impossible. Although we *have* chosen a limited list as it facilitates this research, we do not necessarily believe that an exhaustive list would be possible. Normatively this may well prove problematic; for this study, we are concerned with describing what is rather than with what ought to.

## Conclusion

This study aims to be part of answering the recent call for research in aid of understanding practices of contextualisation. As healthcare coverage decisions are a particularly fruitful area to study these practices in, we have compared the use of contextual factors, defined as situation-specific considerations, in documentations that provide a justification, or rationale, of these decisions as offered by HCKC/NIHDI (Belgium), NICE (England), G-BA (Germany), and ZIN (the Netherlands). To study these, we employed group interviews with 3 national experts per institute, document and web site analysis, and a workshop with 1 to 2 experts per country (together part 1), and the analysis of 4 different case studies across these 4 countries, which varied greatly in terms of type of technology (part 2). From this data set, we conclude that these 4 national healthcare institutes all utilise situation-specific contextual factors in their decision documents. These contextual factors are employed ‘around the table,’ that is, established in deliberation. Though some may remain implicit, others are not only explicated, but *actively integrated* in the decision documentation, thus strengthening the decision by making it more sensitive to the case at hand. Moreover in this data set, there are strong similarities in terms of how these contextual factors are used: similar patterns of contextual factor use lead to similar decisions in different countries. These observations do not use context as non-specific explanatory resources, as critiqued by Asdal in particular,^26,27^ but instead focus on the people and their processes required to actively integrate these considerations. It also calls for future research on patterning of these contextual factors in deliberative settings. Not all decisions are taken ‘around the table,’ however. Half the decisions were taken or retaken in another setting, with the documentation to back up the final decision sometimes completely absent. We note this may impact the legitimacy of these decisions, and call for future research efforts on how this may affect the daily practice of coverage decision-making.

## Acknowledgements

This work benefited from the extensive input of Raf Mertens, former head of Federaal Kenniscentrum voor de Gezondheidszorg, Belgium. This work was supported by Renske Taks, MSc student Health Economics, Policy and Law at Erasmus University Rotterdam, Rotterdam, the Netherlands. An earlier draft of this study was discussed at the Health Care Governance group at the same institution. It was also discussed at the Research colloquium Critical and Interpretative Public Administration, Radboud Universiteit Nijmegen, Nijmegen, the Netherlands, by Dr. Eva Wolf (Tilburg University, Tilburg, the Netherlands). We would like to thank all those mentioned as well as our interviewees for their generous contributions.

## Ethical issues

An ethical waiver, code MEC-2017-539, has been granted for this research by the Medical Ethics Committee (in Dutch: Medisch Ethische Toetsings Commissie, METC) at the Erasmus Medical Centre, Rotterdam, the Netherlands. This waiver indicates that no formal ethical review has been deemed necessary, because the research contained no medical-scientific research question and the interviewees have not been subjected to a treatment or behavioural intervention. Under the research protocol as submitted to the METC, interview audio files and transcripts are kept confidential, and interviewees are anonymised in the manuscript.

## Competing interests

This work was supported by the Dutch National Health Care Institute [grant number 22070000.999.015]. JZ is employed here and was involved in data collection, review, and approval of the manuscript.

## Authors’ contributions

AdB, BB, and TKV designed part 1 of the study. BB, JZ, and TKV conducted the semi-structured group interviews. TKV analysed the interviews and supplementary data and prepared the presentation on the data collected so far for the workshop at the end of part 1, which was attended by all authors. At this workshop, the collective decision was made to extend the research to part 2, with design and criteria formulation a collective effort. JZ helped identify the cases, and TKV (aided by Renske Taks) collected and analysed the data, and wrote up the final manuscript, directly supported by AdB and BB. All authors approved the final manuscript.

## Authors’ affiliations

^1 ^Erasmus School of Health Policy & Management, Erasmus University Rotterdam, Rotterdam, The Netherlands. ^2^National Institute for Health and Care Excellence (NICE), London, UK. ^3^Federal Joint Committee (Gemeinsamer Bundesausschuss), Berlin, Germany. ^4^Erasmus School of Social and Behavioural Sciences, Erasmus University Rotterdam, Rotterdam, the Netherlands. ^5^National Health Care Institute (Zorginstituut Nederland), Diemen, The Netherlands.

## Supplementary files


Supplementary file 1. Working Procedures Per Country.

Supplementary file 2. Interview Guide for Semi-structured Interviews.

## Key messages

**Implications for policy makers**

- Situation-specific contextual factors feature in many healthcare coverage decisions; these decisions are not made based on criteria alone.
- Making healthcare coverage decisions requires expertise both in terms of contributing such contextual factors and making them part of the decision.
- Despite apparent differences in level of formalisation of these contextual factors, countries in Western Europe appear to use similar contextual factor patterns. This may provide reasons for collaboration or exchange on more qualitative aspects of decision-making.

**Implications for the public**

Those who make decisions on what should, and what should not, be provided in terms of healthcare (through a basic benefits package or national formulary) use considerations that vary per decision in doing so. These considerations make decisions more sensitive to the specifics of the type of healthcare they are deciding on and to the patients that hope to be helped by this technology. These considerations thus make the decision and its justification, or rationale, stronger. This research shows that there are similarities between decisions made in Belgium, England, Germany, and the Netherlands, even when looking at decisions that vary a lot in terms of type of technology. This insight may aid decision-makers to make better decisions.
