# Supplementary material for: Around the Tables – Contextual Factors in Healthcare Coverage Decisions Across Western Europe
Source: Int J Health Policy Manag. 2020 Jan 19;9(9):390–402. doi: 10.15171/ijhpm.2019.145 (PMC7557427; doi:10.15171/ijhpm.2019.145)
Supplement: Supplementary file 1 — Working Procedures Per Country. [file ijhpm-9-390-s001.pdf]

Supplementary file 1. Working Procedures Per Country

The Netherlands

The Dutch healthcare system is insurance-based: all Dutch citizens are obliged to take out health insurance with a private insurance company. However, the bare minimum for which they are ensured, the basic benefits package, is the same for all citizens and is established by the government. The Dutch Healthcare Institute (ZIN) advises the Minister of Health in his or her decision as to what should, and should not, be covered.[1] The mission of the Institute is to provide "no less than needed, but no more than necessary" healthcare (as shown on their website, accessed January 17, 2019), a mission that is worked out through advice on the basic benefits package, but also, for example, setting of quality standards.

ZIN employs four *a priori* established criteria, also called "package principles": effectiveness and cost-effectiveness of the technology, feasibility of coverage (including budget impact), and necessity, which includes individual burden of disease and necessity of coverage. The process at the Institute is guided by Institute employees, who also write the final advice document and all previous drafts. The process starts with a scoping session with stakeholders, like patient organisations, healthcare providers, pharmaceutical companies, and health insurers, who are invited to comment on the process. The scoping is followed by an extensive examination of the scientific evidence in the Scientific Advisory Council. The third step is the appraisal, or contextualisation, of this evidence in the Advisory Committee Package in a setting that is open to the public. Generally, some of the stakeholders will be present here as well, and there is an opportunity to contribute to the deliberations, which ought to consider wider societal values pertaining to the coverage. The final advice is then formulated and approved by the Board of Directors, before it is sent to the Minister.[2,3]

Belgium

Belgium, similar to the Netherlands, operates on a social health insurance system basis, which mandates citizens to take out basic health insurance with an insurer. These insurance organisations, called mutualities, operate on a not-for-profit basis. Decisions on what is covered by this compulsory health insurance are discussed at the National Institute for Health and Disability Insurance (NIHDI). NIHDI allocates the national healthcare budget, sets standard prices for treatments, and inspects the mutualities, supervised by the Minister of Public Health and Social Affairs. The Health Care Knowledge Centre (HCKC), in contrast, is responsible for research and scientific advice concerning the basic health insurance, taking on a consultancy role for policy makers, who may commission such research.[4] This research generally takes a multi-disciplinary approach. HCKC counts over 45 experts with a background in medicine, economy, sociology, law, and/or ethics. They formulate recommendations together, aided where necessary by external parties, and in collaboration with the Board of Directors.[5] The coverage decision proposals of NIHDI are sent to the Minister who makes the final decision.

England

The NHS, the National Health System in the United Kingdom, is publicly funded, free at the point of delivery, based on clinical need, not the ability to pay, and is aimed at meeting the needs of everyone.[6] The National Institute for Health and Care Excellence (NICE) provides guidance and advice concerning healthcare services in England.

NICE was established in 1999 in an attempt to tackle so-called postcode lottery in access to new technologies. The Health and Social Care Act of 2012 notes that developing its advice or guidance, NICE must have regard to the broad balance between the benefits and costs of the provision of health services or of social care in England, the degree of need of persons for health services or social care in England, and the desirability of promoting innovation in the provision of health services or of social care in England. The NICE single technology appraisal is the primary, standardised way NICE evaluates old and new forms of care, but also multiple technology appraisals and fast track appraisals are available. Moreover, NICE's guideline programme considers the care and services suitable for most people with a specific condition or need, and people in particular circumstances or settings.[7]

The starting point for technology appraisals is the evidence submitted by the company, where when it comes to a guideline, NICE uses academic collaborating centres to bring together the evidence. In technology appraisals, this evidence is then considered by the ERG, and added to it evidence from external parties, which may include clinical specialists, commissioning experts and patient experts. The appraisal committee subsequently convenes to appraise the available evidence in terms of clinical and cost-effectiveness, consider advice given by NICE, board, and drawing on social value judgements, including those informed by the Citizens Council, to come to a first Appraisal Consultation Document (ACD) containing preliminary recommendations.[8] This Citizens Council, which NICE has had for many years, helps frame the social value judgements that the independent committees are asked to consider when formulating NICE guidance. Once it is published, all stakeholders, including the general public, may comment on the ACD, which are potentially taken along in NICE’s final recommendation.[9]

Germany

The healthcare system in Germany is social insurance based, like the Netherlands and Belgium, with the statutory health funds (SHFs) covering circa 90 % of the German population, and private insurance covering the rest. The costs for insurance are shared between employers and employees, and German citizens are free to choose among the SHFs.[10] All SHFs are represented in the National Association of Statutory Health Insurance Funds (GKV) - Spitzenverband, the SHF umbrella organisation. The Gemeinsamer Bundesausschuss (G-BA) is the highest, independent organisation in the German healthcare system, playing a major role in the decision-making process concerning which services are covered by the SHFs and issuing directives concerning the national benefits package. It brings together four major organisations representing physicians, dentists, hospitals, and insurance funds respectively. The German Social Code, Book 5 (SGB-V) sets out the lawful responsibilities of G-BA, specifying rules for reaching agreement, appointing members and involving patients and third parties.

The main decision-making body of G-BA, the plenum, is a deliberative setting in which decisions are reached concerning which types of care are in- or excluded. The plenum comprises thirteen voting members and five patient representatives. Of these thirteen voting members, three are impartial (including the chair), five are representatives from the GKV-Spitzenverband, and the other five are care provider representatives. Cases are prepared by one of the nine subcommittees for discussion in the plenum, which have their own expertise.[11] Cost-effectiveness data in particular are prepared by Institut für Qualität und Wirtschaftlichkeit im Gesundheidswesen

(IQWiG), the independent federal organisation set up to evaluate medical efficiency, quality and effectiveness of treatments. These data are considered in the plenum next to consultations by experts and practitioners.

References

1. Couwenbergh B, van der Meer F, Weghaus-Reus S, Schelleman H, Zwaap J. *Pakketbeheer in de praktijk deel 3*2013.
2. Zorginstituut. *Pakketadvies in de praktijk: wikken en wegen voor een rechtvaardig pakket*2017.
3. Zorginstituut. *Reglement Adviescommissie Pakket.* https://www.zorginstituutnederland.nl/over-ons/publicaties/besluit/2016/12/05/reglement-adviescommissie-pakket-zorginstituut-nederland. (Accessed 14 September 2017). 2016.
4. Centre BHCK. *Annual Report 2017.* https://annualreport.kce.be/2017/en/annual-report-2017/2017.
5. Centre BHCK. Our team. 2019.
6. Grosios K, Gahan PB, Burbidge J. Overview of healthcare in the UK. *EPMA Journal.* 2010;1(4):529-534.
7. NICE. *Technology Appraisal Processes - CDF.* https://www.nice.org.uk/Media/Default/About/what-we-do/NICE-guidance/NICEtechnology-appraisals/process-and-methods-guide-addendum.pdf. 2016.
8. Dakin H, Devlin N, Feng Y, Rice N, O'Neill P, Parkin D. The Influence of Cost-Effectiveness and Other Factors on Nice Decisions. *Health Economics.* 2015;24(10):1256-1271. doi:10.1002/hec.3086
9. NICE. Guide to the methods of technology appraisal. 2013. Accessed 19 September 2019.
10. ISPOR. *Pharmaceutical HTA and Reimbursement Processes - Germany.* https://www.ispor.org/HTARoadMaps/Germany.asp. 2009.
11. Bundesausschuss G. The Federal Joint Committee: who we are and what we do. Accessed 19 September 2019.
