# Supplementary material for: Around the Tables – Contextual Factors in Healthcare Coverage Decisions Across Western Europe
Source: Int J Health Policy Manag. 2020 Jan 19;9(9):390–402. doi: 10.15171/ijhpm.2019.145 (PMC7557427; doi:10.15171/ijhpm.2019.145)
Supplement: Supplementary file 2 — Interview Guide for Semi-structured Interviews. [file ijhpm-9-390-s002.pdf]

**Supplementary file 2.** Interview Guide for Semi-structured Interviews

• Please tell us about yourself, your institute's coverage decision-making process, its institutional setting, etc. (15 min. presentation by interviewees, prepared beforehand)

• What is your general impression of how necessity and its related notions (eg, need and situation of the patient and the responsibility of society) are used in your country? Could you give one or two examples of a decision where necessity was important and/or decisive?

• In your opinion, is there anything that could be improved in decision-making processes based on necessity in your country? Could you give an example of a decision for which this would have made a difference?

• What are your thoughts on the finding that these necessity argumentations may vary in usage and validity? Is there anything that resonates with your experience particularly? Could you give examples?

• To what degree are these findings understandable and relevant for your work? How would they impact future decisions?

• What (type of) research outcomes would be most valuable for your work in terms of this study? How do you think they might be used at your workplace?

• What it has been like to participate in this interview, what has been good, and what could be improved upon?

• Is there anything else you would like to share with us before we conclude the interview?
